# Improving Coronary Artery Disease Diagnosis in Cardiac MRI with Self-Supervised Learning

**DOI:** 10.3390/diagnostics15202618

**Published:** 2025-10-17

**Authors:** Usman Khalid, Mehmet Kaya, Reda Alhajj

**Affiliations:** 1 Department of Computer Engineering, Firat University, Elazig 23200, Turkey; 2 Department of Computer Science, University of Calgary, Calgary, AB T2N 1N4, Canada; 3 Department of Computer Engineering, Istanbul Medipol University, Istanbul 34810, Turkey; 4 Department of Health Informatics, University of Southern Denmark, 5000 Odense, Denmark

**Keywords:** self-supervised learning, supervised pretext, unsupervised pretext, out-of-distribution, adversarial attack

## Abstract

**The Background/Objectives:** The excessive dependence on data annotation, the lack of labeled data, and the substantial expense of data annotation, especially in healthcare, have constrained the efficacy of conventional supervised learning methodologies. Self-supervised learning (SSL) has arisen as a viable option by utilizing unlabeled data via pretext tasks. This paper examines the efficacy of supervised (pseudo-labels) and unsupervised (no pseudo-labels) pretext models in semi-supervised learning (SSL) for the classification of coronary artery disease (CAD) utilizing cardiac MRI data, highlighting performance in scenarios of data scarcity, out-of-distribution (OOD) conditions, and adversarial robustness. **Methods:** Two datasets, referred to as CAD Cardiac MRI and Ohio State Cardiac MRI Raw Data (OCMR), were utilized to establish three pretext tasks: (i) supervised Gaussian noise addition, (ii) supervised image rotation, and (iii) unsupervised generative reconstruction. These models were evaluated against  Simple Framework for Contrastive Learning (SimCLR), a prevalent unsupervised contrastive learning framework. Performance was assessed under three data reduction scenarios (20%, 50%, 70%), out-of-distribution situations, and adversarial attacks utilizing FGSM and PGD, alongside other significant evaluation criteria. **Results:** The Gaussian noise-based model attained the highest validation accuracy (up to 99.9%) across all data reduction scenarios and exhibited superiority over adversarial perturbations and all other employed measures. The rotation-based model exhibited considerable susceptibility to attacks and diminished accuracy with reduced data. The generative reconstruction model demonstrated moderate efficacy with minimal performance decline. SimCLR exhibited strong performance under standard conditions but shown inferior robustness relative to the Gaussian noise model. **Conclusions:** Meticulously crafted self-supervised pretext tasks exhibit potential in cardiac MRI classification, showcasing dependable performance and generalizability despite little data. These initial findings underscore SSL’s capacity to create reliable models for safety-critical healthcare applications and encourage more validation across varied datasets and clinical environments.

## 1. Introduction

Self-supervised learning (SSL) has proven highly beneficial in various domains, particularly in healthcare. One of the key challenges in SSL is designing an effective pretext model tasked with acquiring valuable feature representations from unlabeled data. These learned features can then be transferred to downstream tasks, such as disease classification. A well-designed pretext model is critical in self-supervised learning, as it extracts meaningful information from unlabeled data that can be fine-tuned for downstream tasks. Pretext algorithms can be categorized into supervised and unsupervised approaches, including instance discrimination [1,2], generative, and contrastive learning methods. Before SSL, semi-supervised and transfer learning were commonly used to reduce reliance on labeled data, particularly in healthcare, by leveraging features from large-scale datasets such as ImageNet. SSL has gradually shifted focus toward learning from unlabeled data through pretext tasks, offering an alternative to traditional transfer learning. Studies comparing SSL and traditional supervised learning report mixed results for accuracy, highlighting the importance of assessing overall model performance, including out-of-distribution scenarios and adversarial attacks [3,4,5,6,7].

The effectiveness of an SSL framework heavily depends on the pretext model, making its design a crucial area of research. Pretext algorithms can be broadly categorized into supervised pretext algorithms and unsupervised pretext algorithms, based on their mechanisms for feature learning. This study evaluates the strengths and limitations of both approaches in healthcare applications. A substantial edge of SSL is its capacity to exploit large-scale unannotated datasets, reducing reliance on costly manual annotation. In healthcare imaging, especially cardiac Magnetic Resonance Imaging (MRI), annotated datasets are scarce due to the expertise and time required for labeling [8]. Computed Tomography (CT) imaging has also emerged as a critical modality for detecting coronary artery disease (CAD), particularly in classification and segmentation tasks. Recent studies, such as the works of [9,10], demonstrate the growing role of CT in cardiovascular diagnosis and highlight the importance of integrating multi-modal imaging in AI research.

Traditional supervised learning approaches depend on annotated data, which limits scalability. In contrast, SSL allows models to learn meaningful representations from raw data using pretext tasks and to be fine-tuned for CAD detection and classification problems. To examine these approaches in practice, this study experiments with one of the most significant challenges in healthcare: coronary artery disease. CAD remains a leading cause of global mortality, significantly contributing to the burden of cardiovascular diseases [11]. Early and accurate detection is critical for improving patient outcomes. Cardiac MRI has become an essential tool for diagnosing CAD by identifying structural and functional abnormalities in the heart. However, the high-dimensional and complex nature of medical imaging data poses challenges for traditional diagnostic approaches, creating the need for innovative AI-driven solutions.

Researchers have proposed several deep-learning architectures, including AlexNet [12], but supervised learning methods still face limitations due to their dependence on large annotated datasets and lack of generalized pre-trained models for medical applications. SSL addresses this gap by learning robust feature representations from unlabeled data, which can then be fine-tuned with minimal labeled data to improve model generalization and performance [8,13]. Within this context, this research experiments with categorizing previously explored SSL approaches based on their mechanisms, while formulating general mathematical pseudo-expressions to clarify their theoretical foundations. It validates these algorithms experimentally within their respective categories, highlighting their strengths and limitations. The study develops a supervised pretext algorithm for CAD classification that is transferable across downstream tasks in healthcare diagnostics. A comparative analysis is conducted under data reduction scenarios, evaluating performance, accuracy, and against out-of-distribution data and adversarial attacks.

The contribution extends beyond existing SSL literature by integrating a supervised pretext model using Gaussian noise and by demonstrating its real-world applicability and resilience in clinical tasks under data scarcity and adverse conditions. Finally, unlike prior SSL studies that rely mainly on generic datasets such as ImageNet or CIFAR, this research applies SSL directly to clinically relevant MRI images, focusing on coronary artery disease detection in an exploratory setting. We clarify that, in clinical practice, CAD diagnosis typically relies on coronary angiography or CT angiography, and cardiac MRI is not the gold standard. The study addresses challenges such as label scarcity, adversarial robustness, and out-of-distribution generalization. While our bi-nary classification is a simplification, experiments conducted on MRI data provide insights that are novel, empirically validated, and practically valuable, highlighting the feasibility of SSL for CAD detection and paving the way for future multi-class, clinically validated studies.

## 2. Materials and Methods

### 2.1. Supervised Pretext and Unsupervised Pretext Algorithms

The design of pretext models, a critical component in self-supervised learning, can be broadly classified into two main categories: supervised and unsupervised pretext models. This classification is based on the methodology employed to train the pretext model, with each category offering distinct approaches and advantages. In a supervised pretext model, the training process mirrors traditional supervised learning, where labeled data is used to guide the model’s learning. However, it is important to note that the labels in supervised pretext models differ from those used in downstream tasks. Specifically, the labels utilized in the pretext model are artificial (pseudo-labels), generated by employing data augmentation techniques that are not directly tied to the downstream tasks but rather aim to train the model on data transformations. One common approach in supervised pretext models involves data augmentation techniques, such as rotating the original data. These rotations allow the model to learn variations in the same underlying data, enhancing its ability to generalize. After applying these transformations, labels are assigned to both the original and augmented (rotated) data. By doing so, the model is forced to learn features from the augmented data, effectively differentiating between the original and transformed images. This technique has been extensively used in various research studies, such as those aimed at classifying cocoa diseases [14], where the task was to identify disease-related features in cocoa plants by leveraging augmented data.

Furthermore, supervised self-supervised pretext algorithms can also be framed as self-predictive tasks. In such models, the goal is not merely to predict the label of an image but to predict certain transformations or features of the data. The idea of self-prediction in self-supervised learning has gained significant attention, as it allows the model to learn by predicting aspects of the data that are not explicitly labeled. In many cases, these pretext tasks are framed as classification problems, where the model is tasked with distinguishing between the original and the transformed data. This concept of self-prediction addresses key challenges in the availability of labeled data, as it allows the model to learn useful representations without relying heavily on annotated datasets. Previous works in the literature, such as the study conducted by [15], have employed the self-prediction paradigm to address these challenges and improve model performance, particularly in domains where labeled data is scarce.

**Figure 1:**
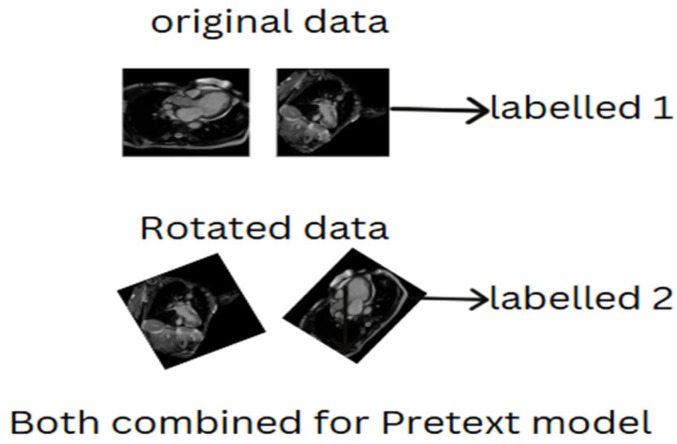
Supervised pretext model formulation from data sample.

To further illustrate the concept of supervised pretext models, Figure 1 provides a visual representation of the approach. The figure shows the original data (which represents unlabeled data) alongside its corresponding rotated version, demonstrating the use of data augmentation as part of the pretext task. This augmented data is assigned a pseudo-label, and the model is tasked with differentiating between the original data and its transformation, such as the rotation applied in this case. The rotation serves as an example of the types of transformations that can be used in supervised pretext models to teach the model invariant features.

**Figure 2:**
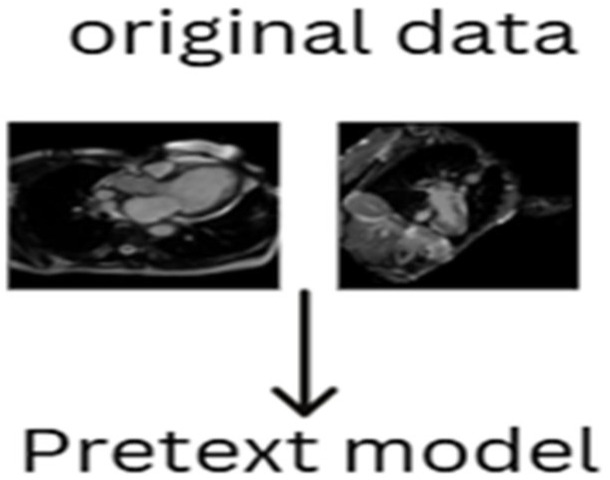
Unsupervised pretext model formulation from data sample.

Table 1 presents a comprehensive summary of various literature sources that have utilized pseudo-labels in the generation of pretext models. This table highlights the diversity of applications and methodologies in which pseudo-labels have been successfully employed, demonstrating their potential for enhancing the learning process in self-supervised models. Conversely, pretext algorithms that do not require labels during the training process are considered unsupervised pretext models or algorithms. These unsupervised methods take a different approach by allowing the learning process to be entirely label-free. In unsupervised pretext models, the algorithm is not provided with any explicit labels but instead learns directly from the inherent structure of the data. The absence of labels means that the model is tasked with finding patterns, features, or representations that are useful without the guidance of any predefined outputs. These approaches can extract meaningful features from the data based purely on its structure and intrinsic properties, often relying on techniques like clustering, contrastive learning, or autoencoding, where the model is encouraged to learn representations that are useful for downstream tasks. Figure 2 illustrates an example of an unsupervised pretext algorithm, where the pretext task is derived directly from the unlabeled original data. This model emphasizes the idea that the algorithm can learn from the data’s natural structure without the need for labels or artificial transformations. The unsupervised approach is particularly advantageous in scenarios where labeled data is limited or unavailable, and it allows the model to learn from large datasets of unlabeled data without the need for costly annotation processes.

Table 2 provides a detailed summary of various studies and literature sources that have utilized unsupervised pretext models or have avoided the use of pseudo-labels in their pretext model generation. This table functions as a reference for comprehending the various strategies and methodologies utilized in unsupervised pretext learning, illustrating the capacity of these models to acquire effective representations independent of labeled data.

### 2.2. Mathematical Representation of Two Approaches

As indicated in the definition of supervised and unsupervised pretext algorithms, pretexts with pseudo-labels are directly categorized as supervised. Formulas (1) and (2) below provides the details of this approach.

(1) $$Psupevised=\frac{1}{N}\sum_{j=1}^{N}L(model\left(X_{j};\theta \right),Y_{j})$$

where $X_{j}$ represents the input data, and $Y_{j}$ indicates the generated labels from the unlabeled dataset, obtained through any selected method. *L* is the loss function used to minimize the gap between the prediction and $Y_{j}$, with *N* the total number of pseud0-labeled, $X_{J}$ as the input data sample and *θ* as the model parameters.

(2) $$Punsupervised=\frac{1}{M}\sum_{j=0}^{m}L\left(model\left(T\left(X_{j}\right);\theta \right),X_{j}\right)$$

where $T(X_{j})$ represents the transformation applied during the pretext model. $model\left(T\left(X_{j}\right);\theta \right)$ as the output model to reconstruct *L* as the loss function between the prediction and original, and $X_{J}$ and *M* as the total samples.

### 2.3. Dataset and Pre-Processing

**Figure 3:**
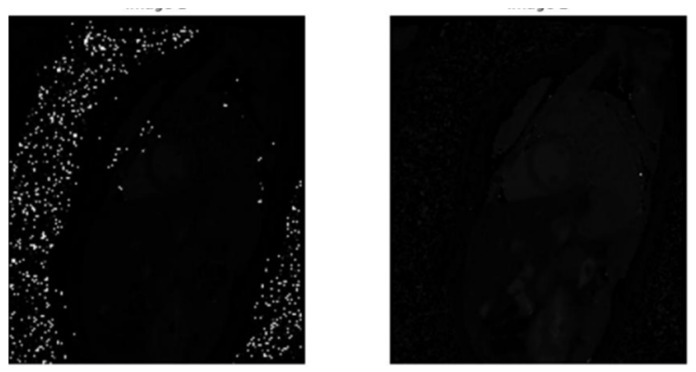
Manually removed dataset.

The first dataset used in this study, sourced from [24], is particularly well-suited for deep learning applications due to its large size, rich content, and diversity. It includes more than 2 GB of MRI scans, which encompass both healthy individuals and patients diagnosed with coronary artery disease (CAD). This dataset offers a wide array of information, enabling the development of various pretext tasks and algorithms. These pretext tasks, once designed, can be applied to downstream tasks, thereby enhancing the model’s ability to learn robust and transferable features for a variety of related applications. A total of 19,036 MRI images (evenly split between healthy and CAD cases) were allocated for the pretext phase, while 2517 healthy and 2517 CAD MRI images were allocated for the downstream task. The dataset is available in different sizes, but a size of 180 × 180 × 3 has been commonly adopted throughout the literature due to its balance between computational efficiency and the ability to retain sufficient image details. Prior to model training, the dataset underwent both manual and computational preprocessing to ensure high data quality. In the manual phase, corrupted or clinically irrelevant images (e.g., low contrast or missing structures) were visually inspected and removed, as illustrated in Figure 3.

In the computational cleansing, a combination of median and Gaussian filters was applied to reduce common imaging noise. A median filter was used to effectively suppress salt-and-pepper noise, which appears as isolated white and black pixels in MRI scans and can obscure fine details of the data. This was implemented using OpenCV’s cv2.medianBlur(image, 3) function. Additionally, some images exhibited Gaussian noise, typically introduced during image acquisition or compression, which can lead to pixel inconsistencies across smooth regions. To address this, a Gaussian blur filter was selectively applied using cv2.GaussianBlur(image, (3, 3), 1.2), which smooths local intensity variations without significantly blurring anatomical boundaries. The decision to use both filters was based on visual inspection and experimentation to ensure that true features were preserved while noise was effectively removed. Each image was dynamically passed through either or both filters, depending on its detected noise characteristics, allowing for a flexible and adaptive pre-processing pipeline.

Beyond denoising, all image pixel values were normalized to the range [0,1] to ensure consistent input distributions for neural network training. This normalization improves convergence and numerical stability during optimization. These preprocessing steps—filtering, normalization, and manual inspection—were essential to ensure that the data used for both pretext and downstream tasks was clean, high-quality, and reflective of the true underlying anatomical structures, thereby improving the model’s ability to learn meaningful and generalizable features.

The Ohio State Cardiac MRI Raw Data (OCMR), the other dataset used in this study dataset, is a publicly available resource designed to support research in cardiac imaging and reconstruction [25]. It includes raw k-space and image data acquired from patients with a variety of cardiac conditions, collected using multiple MRI scanners and protocols. OCMR provides a standardized benchmark for developing, testing, and comparing advanced methods in image reconstruction, quantitative analysis, and machine learning applications in cardiovascular MRI. It comprises 53 fully sampled and 212 prospectively under sampled cine series (totaling 81 slices) collected across multiple scanners and protocols.

### 2.4. Pretext Algorithms

The most critical aspect of any self-supervised algorithm is the pretext task, which serves as the foundational step in the learning process. The pretext task occurs after the acquisition of an unlabeled dataset and plays a vital role in driving the learning of useful features without the need for manually labeled data. In this phase, a model is trained on a task designed to extract meaningful representations from the data, which can later be transferred to more specific downstream tasks, such as classification or segmentation. The robustness and success of any self-supervised algorithm are heavily dependent on the quality of its pretext model. A well-designed pretext model allows the algorithm to learn generalized features that are not only relevant to the pretext task but also applicable to various real-world applications. If the pretext task is poorly chosen or does not capture essential patterns within the data, the model may fail to learn meaningful features, resulting in poor performance when applied to downstream tasks. Thus, the careful selection and design of the pretext model are crucial for ensuring the algorithm’s effectiveness in learning useful representations and achieving successful transfer learning. A total of 19,036 (9518 × 2) samples, comprising original data and pseudo data (including Gaussian noise and augmented images), were used throughout the pretext phase. From this dataset, 7616 samples were used to evaluate the performance of the pretext models, as reflected in the respective confusion matrices. The remaining data were split into 80% for training and 20% for validation the pretext model.

#### 2.4.1. Selection of Pretext Tasks

This study employs three pretext tasks—Gaussian noise, image rotation, and generative reconstruction—based on their common usage and relevance in existing self-supervised learning (SSL) literature. Generative models and contrastive approaches are among the most widely used techniques in SSL, and thus, a generative method is included to represent that category. Similarly, self-predictive strategies such as applying data augmentations or distortions to the original input to create pseudo-labels are also commonly adopted; Gaussian noise and image rotation are used in this study to reflect that practice. To provide a strong benchmark comparison, SimCLR is incorporated as it represents a popular and well-established contrastive SSL framework. The combination of these approaches allows for a comprehensive evaluation across supervised and unsupervised SSL paradigms in the context of medical image analysis.

#### 2.4.2. Self-Predictive Pretext

**Figure 4:**
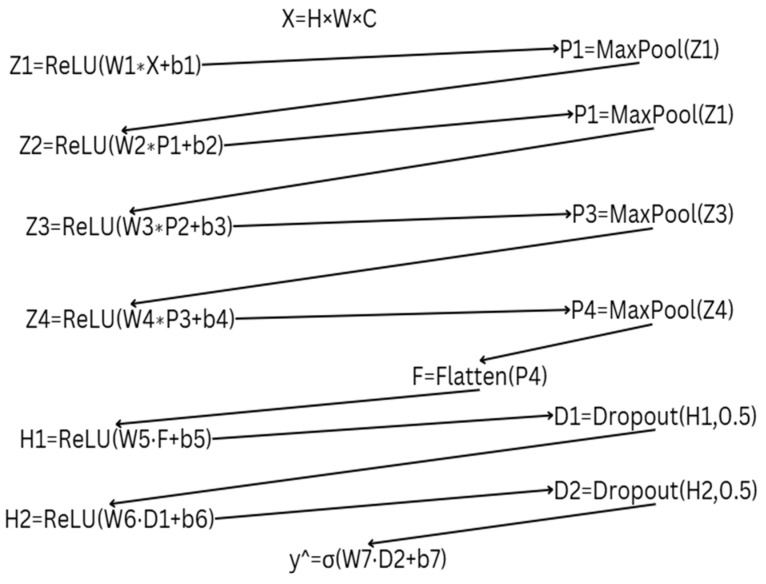
Details of the Pretext Model.

Self-predictive pretext tasks involve generating pseudo-labels for the original dataset, thereby creating a new dataset with corresponding pseudo-labels. This approach allows the model to learn meaningful representations without relying on manually annotated labels. The pretext model is trained to perform either classification or regression based on these pseudo-labels, enabling it to extract essential features from the data. Through this process, the model captures inherent patterns and structures within the dataset, improving its ability to generalize to downstream tasks. The self-predictive pretext models developed using this approach are categorized under supervised pretext algorithms, as they involve training with predefined pseudo-labels. These models play a crucial role in enhancing feature learning, ultimately contributing to improved performance in subsequent tasks such as disease classification, segmentation, or anomaly detection in medical imaging. All pretext algorithms were trained on CNN models along with their corresponding downstream tasks. Details of the pretext CNN algorithms can be seen in Figure 4. All self-predictive pretext models were trained on MRI images resized to 180 × 180 pixels. A consistent batch size of 32, 10 training epochs, and the Adam optimizer with a learning rate of 1 × 10^−4^ were used across all models. Binary cross-entropy was employed as the loss function since the task is a binary classification problem (i.e., distinguishing between original and noised data). Specifically, for the Gaussian noise pretext task, a noise factor of 0.01 was applied to the original images to generate the pseudo data used for training. All models were compiled using binary cross-entropy loss and evaluated using accuracy as the primary metric.

Z represent the convolutional layer, with Z1 referring to Convolutional Layer 1, and so on. The asterisk (*) represents the operation within each convolutional layer. After each convolution, a max-pooling operation is applied, denoted as P1 for the first pooling layer, P2 for the second, and so on. F represents the flatten layer, followed by fully connected dense layers, denoted as H. The final layer, y^, represents the output of the CNN model, where σ is the sigmoid function, which outputs either 0 or 1.W1 represents the weights for the convolutional layers through W4, and the corresponding biases are b1 through b4. The weights for the fully connected layers are represented by W5 and W6, with biases b5 and b6, respectively. W7 and b7 correspond to the weights and bias for the output layer. The input layer X has dimensions H (height) = 180, W (width) = 180, and C representing the number of channels (3 for RGB images).

1. Introduction of Gaussian Noise to the Original Data

Gaussian noise is introduced into the original image to create a perturbed version. The added noise is small enough that it is imperceptible to the human eye, making it difficult to distinguish between the original and the perturbed image, and ensuring that the noise does not drastically alter the visual appearance. The goal of this process is to simulate real-world noise that may naturally occur in imaging systems, thereby allowing the model to learn robust features that are resilient to such noise. The original image is assigned a label of 0, while the noisy version of the image, which the Gaussian noise has modified, is assigned a label of 1.

This setup creates a binary classification problem, where the model is tasked with distinguishing between the original image (label 0) and the perturbed image (label 1). By training the model to classify these two types of images, it learns to identify subtle differences between the original and noisy versions, enhancing its ability to generalize to various perturbations that may appear in real-world data.

**Figure 5:**
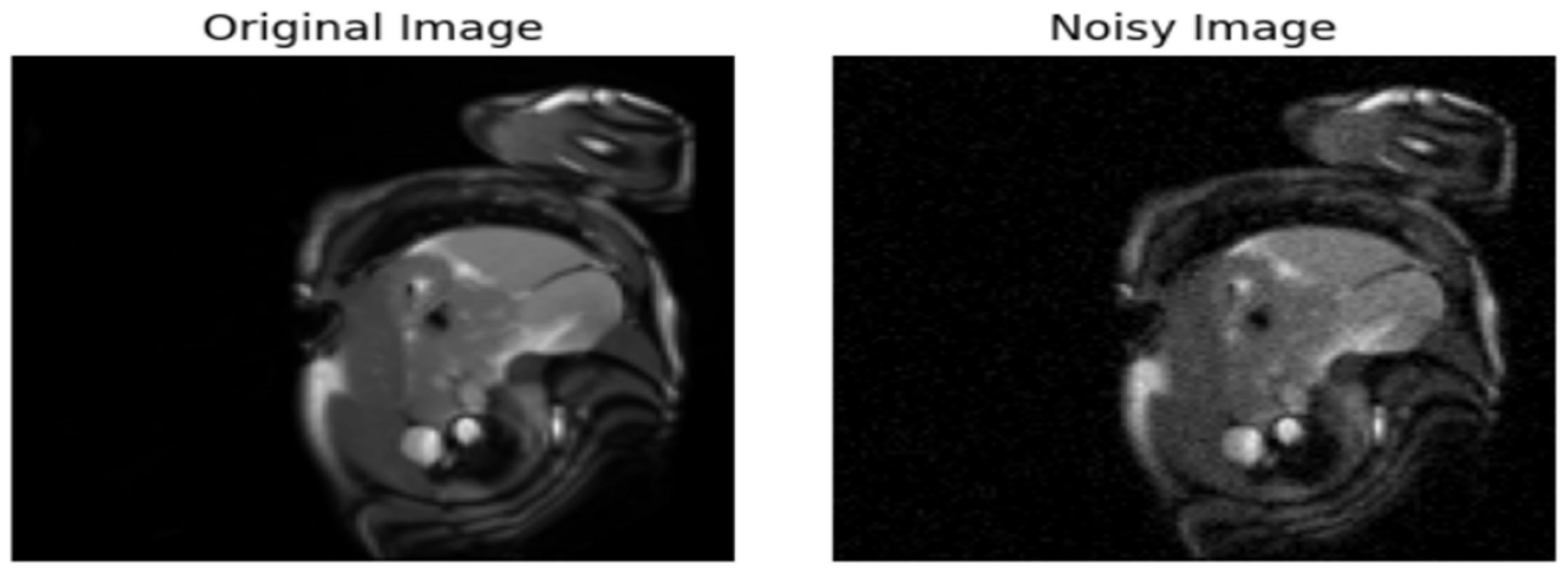
Sample of the Perturbed Image.

In this framework, the model is trained on pairs of images: the original (unmodified) image and its noisy counterpart. The model’s objective is to predict the label of the image, where the label indicates whether the image is the original (0) or the perturbed version. This is typically approached as a binary classification task, where the model’s output is a probability score indicating the likelihood that a given image is the noisy version. Figure 5 illustrates a sample of the applied Gaussian noise.

2. Formulation

Let *t* $X_{0}\in R^{H\cdot W\cdot C}$ denote the original image, where *H*, *W* and *C* are the height, width and channel, respectively. A perturbed version $X_{n}$ is generated by adding Gaussian noise $N\;∼\;N(\mu ,\sigma^{2})$.

Formulas (3)–(6) represent the addition of Gaussian noise, the assignment of pseudo-labels, the binary cross-entropy loss, and a repeated labeling rule, respectively.

(3) $$X_{n}=X_{0}+N\;,\;\;\;N\;∼\;N(\mu ,\sigma^{2})$$

This process creates a binary classification task where the original data *X*_0_ is labeled as *y* = 0, and the noisy image *X_n_* is labeled as *y* = 1.

(4) $$y=\left\{\begin{array}{c} 0,\;\;\;if\;x=X_{0}\\ 1,\;\;if\;x=X_{n} \end{array}\right.$$

where *y* = 0 indicates the original dataset and *y* = 1 represents the noisy dataset.

The model is trained to predict the label y∈{0.1}, given the input data x. The main objective is to minimize the binary cross-entropy (BCE) loss.

(5) LBCE=−[y⋅log(y^)+(1−y)⋅log(1−y^)]

where y^∈[0,1] is the predicted probability that the input image is noisy.

Through this methodology, the model is able to distinguish between images with slight perturbations, which is essential in real-world applications where noise is often present in the data. By leveraging the binary classification setup, the model can also serve as a foundation for more complex tasks that require identifying and processing noisy images, making it an effective approach for training robust models in noisy environments.

3. Augmenting the original images

**Figure 6:**
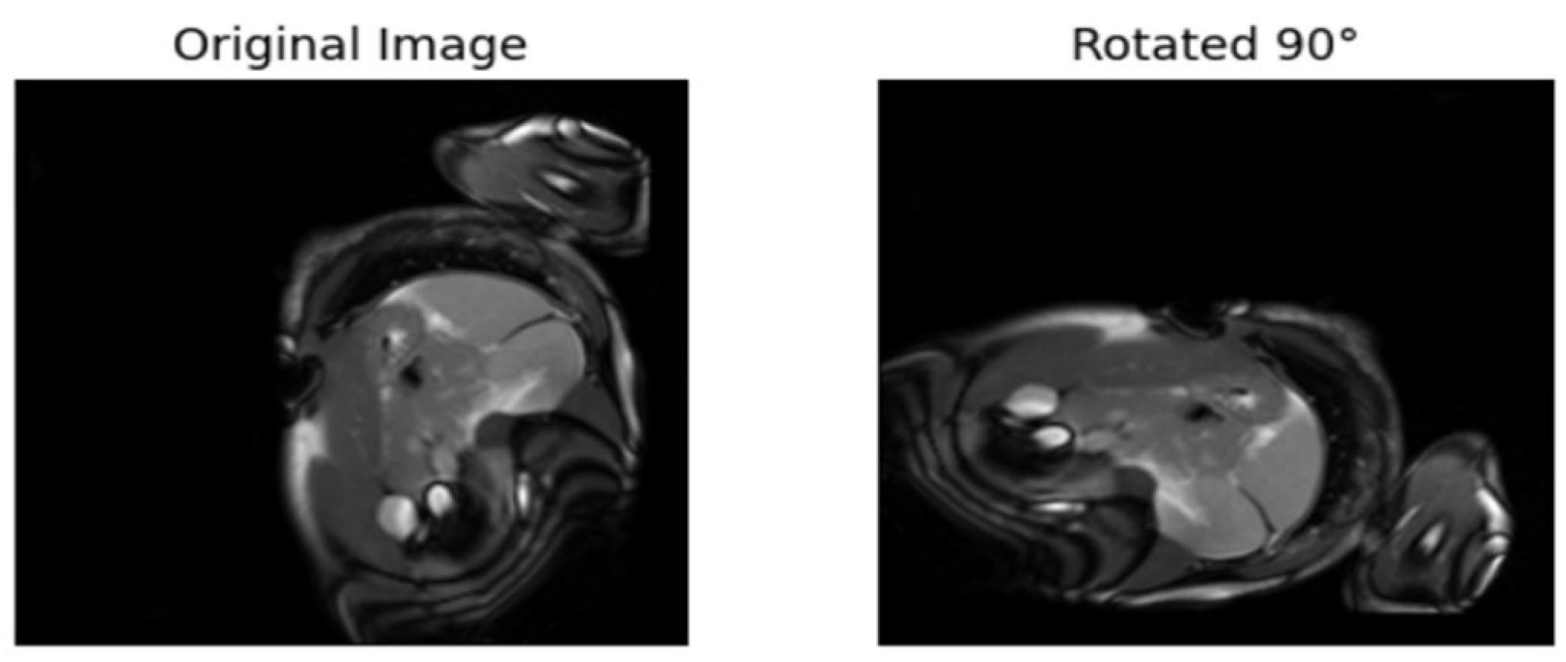
Rotating the original image by 90.

**Figure 7:**
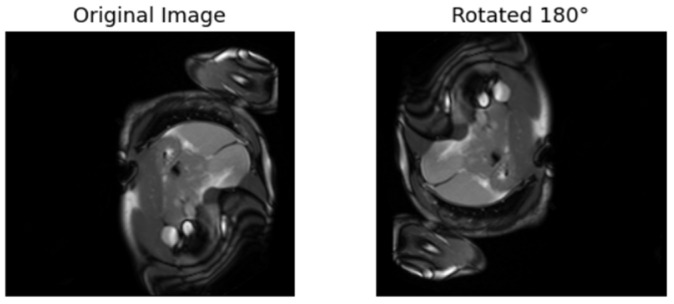
Rotating the original image by 180.

In this section, augmentation is applied to the original images Xo. The original images are randomly rotated by either 90° or 180°, thus distinguishing them from the original images. The original images are labeled as 0, and the rotated images are labeled as 1. The rotated dataset is denoted as Xn. This approach is also considered supervised, as pseudo-labeling is introduced to train the pretext algorithms. Figure 6 and Figure 7 provide examples of the augmented images rotated by 180° and 90°, respectively. This approach has also been employed in other sectors to address classification problems. For instance, the literature in used augmentation to train a pretext model for cocoa disease detection.

(6) $$y=\left\{\begin{array}{c} 0,\;\;\;if\;x=X_{0}\\ 1,\;\;if\;x=X_{n} \end{array}\right.$$

where *y* = 0 is the original image and *y* = 1 is the augmented image and $X_{n}$ the rotated image.

The training of the pretext model under the rotation and that of use of Gaussian noise utilizes the same formula described under Formula (3).

#### 2.4.3. Generative Pretext Model

The concept of generative self-supervised learning is derived from Generative Adversarial Networks (GANs). All pretext tasks in generative self-supervised learning are considered supervised pretext models because no explicit annotation is required during the feature-extraction process. Researchers have proposed various concepts, including masking, which originated in natural language processing (NLP). The idea of masking has been applied to computer vision, where models are trained to predict missing parts or reconstruct hidden regions of an image. Notable literature employing this reconstruction-based approach includes [26].

The study in [27] has highlighted several works based on GANs, including applications in denoising, colorization, and inpainting. Particularly in the healthcare sector, the literature in [28] has reviewed various applications of self-supervised learning (SSL), including its use in predicting PTSD. It also compares SSL-based approaches to the traditional supervised learning methods, demonstrating improvements proposed by other researchers. Features learned through generative self-supervised learning are not limited to classification downstream tasks but are also applicable to segmentation problems in healthcare. For instance, the work in [29] applied generative self-supervision to brain MRI segmentation.

**Figure 8:**
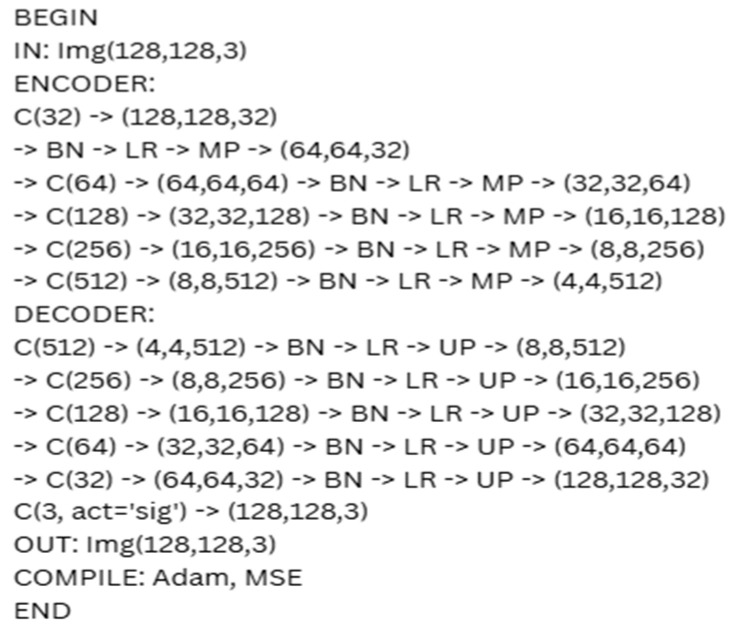
Architecture of the autoencoder.

The pretext task developed through the GAN-based approach involves reconstructing an entire image using an encoder–decoder framework. The focus of this model is not on whether the original data is labeled but, on its ability, to accurately reconstruct the original images. The primary challenge with this approach is minimizing the reconstruction error, typically achieved by using binary cross-entropy. Since the model is trained on original, unannotated images, no pseudo-labels are required. The proposed pseudo-code for this approach is presented in Figure 8. In this figure, C denotes Conv2D with filters and “same” padding, BN signifies BatchNormalization, LR refers to LeakyReLU as the activation function, MP indicates MaxPooling (2 × 2), and UP represents UpSampling (2 × 2). Sig denotes the Sigmoid activation function, Adam represents the Adam optimizer, and MSE signifies the Mean Squared Error, utilized as the loss function.

After the generative pretext model SimCLR model is developed as a base for comparison. During the pretext phase, the SimCLR model underwent training without any labels by creating two distinct augmented versions of each image (flipping, cropping, brightness and contrast adjustments). These versions were processed through a shared encoder (based on AlexNet) to capture valuable image representations. The optimization of the model utilized contrastive loss (NT-Xent), which promotes similarity among features of the same image while distancing the features of different images. This self-supervised method allows the model to acquire significant visual features prior to fine-tuning for the subsequent binary classification task.

**Figure 9:**
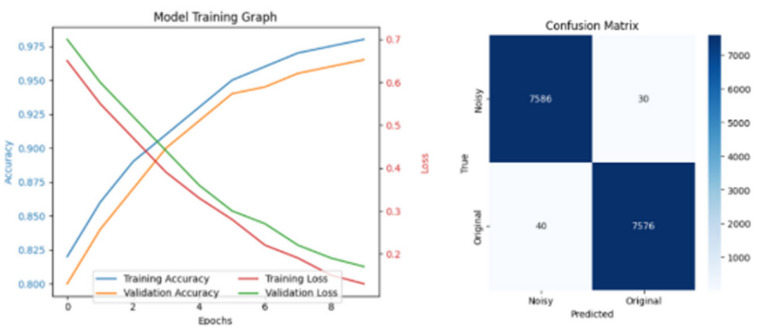
Training and Confusion Matrix from Rotation.

**Figure 10:**
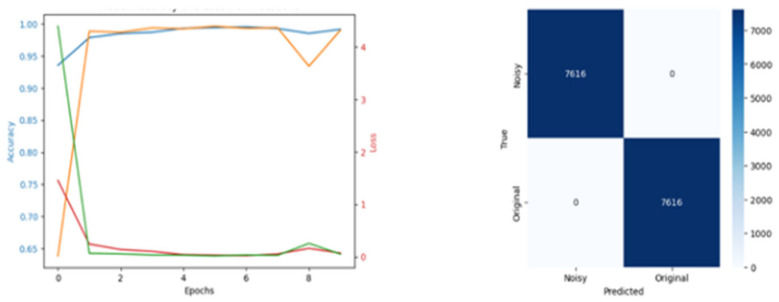
Training and Confusion Matrix from Gaussian Noise.

**Figure 11:**
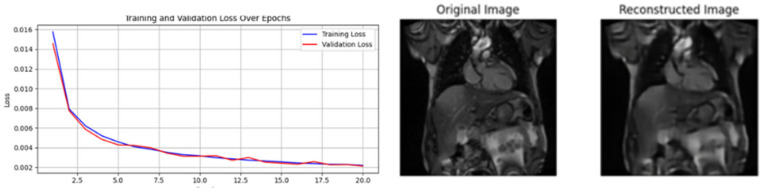
Training Loss and Reconstructed Images from Generative Pretext.

Figure 9, Figure 10 and Figure 11 illustrate the results from the pretext models trained using rotation, Gaussian noise, and generative self-supervised learning, respectively, on the CAD Cardiac MRI dataset.

### 2.5. Downstream Task

A downstream task is considered the end product or final output obtained through the concept of transfer learning. Downstream tasks are not only associated with NLP but also apply to computer vision, where tasks such as classification, segmentation, and anomaly detection can be considered downstream tasks. In natural language processing (NLP), end products such as sentiment analysis, named entity recognition (NER), language translation, and text summarization are achieved using either static or contextualized models, such as Word2Vec and GloVe (static) or BERT and GPT (contextualized). In NLP, several studies have explored this concept, including the works of [30,31].

In this study, the goal is to fully understand which of the two approaches (supervised pretext tasks or unsupervised pretext tasks) is more effective in learning features and serves as a better foundation for comparative analysis. The first three models are obtained directly from individual pretext models (section D, while the last model is obtained from SimCLR as serve as a benchmark in comparing the developed models to a state-of-the-art model.

The downstream task is a binary classification problem, distinguishing between healthy subjects and patients with coronary artery disease (CAD). The downstream model for each task is derived from its respective pretext model, with only the output layer removed, since the input size of 180 × 180 × 3 is consistently maintained across all stages of the study. To adapt the architecture for classification, two dense layers are added, separated by a dropout layer with a rate of 0.5, followed by a final binary output layer.

All downstream models were trained using a batch size of 32, the Adam optimizer, and 20 epochs, with binary cross-entropy used as the loss function. The parameter sizes vary depending on the pretext model used. For the Gaussian noise and rotation-based models, the downstream network contains a total of 35,969,925 parameters, of which 35,576,901 are trainable and the rest are non-trainable. In contrast, the generative pretext-based model has a significantly larger architecture, with 306,881 total parameters, including 35,576,901 trainable and remaining are non-trainable parameters.

## 3. Results

A good model is not solely focused on accuracy; various metrics must be considered. If accuracy were the only factor, traditional supervised models would have the advantage, as indicated by the majority of the literature, such as [7]. Existing studies primarily emphasize accuracy while also investigating the impact of data reduction on the robustness of self-supervised learning (SSL) models. Our study focuses on overall model performance by reducing the CAD Cardiac MRI dataset and comparing supervised and unsupervised approaches in designing a pretext model. This research examines how a robust pretext model influences its downstream task and compares it with a supervised and unsupervised pretext model. Specifically, the dataset used in the downstream task is reduced by 20%, 50%, and 70% to assess which approach yields a more robust model. The total base-labeled dataset consists of 5034 samples. Out of this, 20% is used for validation and 70% for training the remaining 10% for testing the robustness of the models. The dataset reduction follows the same ratio for the training and validation sets. Regarding the FGSM and PGD attacks, a newly introduced image is used for better assessment and comparison. Table 3, Table 4 and Table 5 illustrate the results of using the model trained with Gaussian noise, the pretext model based on rotation, and the pretext model from generative self-supervised learning, respectively. Table 6 represents a general comparison between the three models and SimCLR (Simple Framework for Contrastive Learning of Visual Representations) algorithms trained using the same sample of data. In the context of adversarial attacks, the term ‘no effect’ denotes high robustness to both PGD and FGSM perturbations, ‘less effect’ signifies moderate robustness, while ‘there is an effect’ reflects poor resilience to such attacks.

The pretext model demonstrated the best performance, achieving 100% accuracy on both seen and unseen datasets, which had a significant impact on the downstream task. The reduction in data did not notably affect training or validation accuracy. Regarding adversarial attacks, the model showed minimal sensitivity, as perturbations in the data produced the same outputs as the original data. This robustness can be attributed to the fact that the pretext model was trained using perturbed versions of the original images, giving it an advantage in handling adversarial attacks. With a complete reduction in the base dataset, the model maintained strong accuracy and validation performance. However, a more thorough assessment through additional testing on entirely different datasets revealed that some models trained with less data exhibited slight overconfidence in both adversarial attack scenarios and regarding OOD; the figures presented show the prediction score using a threshold of 50 percent.

The pretext model trained using pseudo-labels derived from the rotation of original images demonstrated lower robustness compared to the other two models—those trained with Gaussian noise and generative approaches. This lower robustness can be attributed to the model’s weaker performance during its pretext training phase. Nevertheless, the model still achieved notable performance in its initial phase, and even with a 20% reduction in training data, it maintained consistent performance across all evaluation metrics. When the training data was reduced by 50%, the model still showed considerable performance in terms of accuracy. However, accuracy alone is not a sufficient measure of robustness. Due to its poor results under adversarial attacks such as Projected Gradient Descent (PGD) and Fast Gradient Sign Method (FGSM), this model is not regarded as a viable option in the context of this study. Therefore, while it provided some useful insights, the literature does not treat it as a robust model.

Pretext tasks from Generative SSL tend to show a reduction in all evaluation metrics as the amount of data decreases. It can be observed that when a moderate amount of data was used, all metrics demonstrated exceptional performance, resulting in a robust model. A 50% reduction in data resulted in only a 0.3% change in OOD detection. Additionally, under both PGD and FGSM attacks, even slight perturbations of the input data significantly affected the model’s predictions, which is a critical concern in the context of medical imaging.

This table shows the results when the training data was reduced by 50%. The Gaussian Noise model performed the best, with 99.9% validation accuracy, high resistance to attacks, and the lowest OOD score (0.04). The Rotation model dropped to 80% accuracy and was easily affected by attacks, with a high OOD score of 0.34. The Generative model had 84% accuracy and showed moderate resistance, while SimCLR reached 97.7% accuracy but also showed only moderate robustness, with an OOD score of 0.29. Overall, Gaussian Noise was the most accurate and robust model when using less data.

Literature comparing Self-Supervised Learning (SSL) and traditional supervised learning highlights differences in terms of accuracy. The work of [7] suggests that if model evaluation is based solely on accuracy, then traditional supervised learning has the upper hand. However, other studies argue the opposite.

## 4. Discussion

In this paper, we argue that with a well-designed pretext model, even traditional supervised learning cannot outperform SSL in terms of accuracy. However, in the healthcare sector, particularly in medical imaging, accuracy alone is insufficient to define a robust model. Other critical factors must be considered, including PGD (Projected Gradient Descent), FGSM (Fast Gradient Sign Method), and Out-of-Distribution (OOD) detection. In particular, OOD detection is crucial, as it allows models to recognize and adapt to newly emerging diseases. A truly robust model should perform well across multiple evaluation metrics, not just accuracy.

Among the pretext models evaluated in this study, the Gaussian noise pretext model, categorized as a supervised approach, demonstrated superior performance with a validation accuracy of 99.9%, showing minimal degradation under adversarial attacks (PGD/FGSM) and a low out-of-distribution (OOD) detection score of 0.04 even with 50% data reduction. This model consistently outperformed the others in terms of both accuracy and robustness. In contrast, the rotation-based supervised model, while achieving a relatively high validation accuracy of 99.0%, exhibited poor robustness beyond 20% data reduction and an elevated OOD score of 0.34, indicating limited reliability under adversarial stress. The generative pretext model, representing the unsupervised category, achieved 96.0% validation accuracy and displayed moderate robustness, although it showed increased sensitivity to input perturbations with an OOD score of 0.30. Comparatively, SimCLR, a widely adopted unsupervised contrastive learning method from the literature, typically reports validation accuracies in the range of 95–97%. While it demonstrates moderate robustness through contrastive feature learning, it remains less resilient without strong augmentations and generally produces higher OOD detection scores (0.10–0.25). Overall, the results underscore that a well-designed supervised pretext task, such as Gaussian noise prediction, can outperform popular unsupervised approaches like SimCLR, particularly in terms of robustness and generalization—key factors in medical imaging applications.

### 4.1. Quantitative Analysis

**Figure 12:**
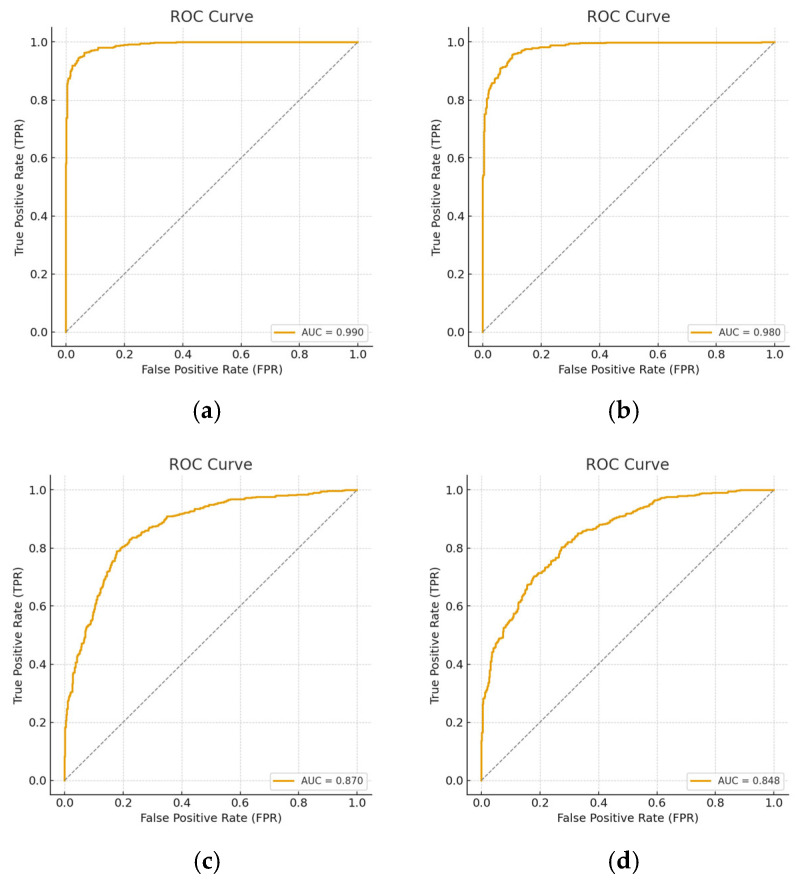
ROC curves for all data reduction for the downstream obtain from rotation pretext model. (**a**) Using all the dataset; (**b**) 20 percent reduction; (**c**) 50 percent reduction; and (**d**) representing 70 percent reduction.

**Figure 13:**
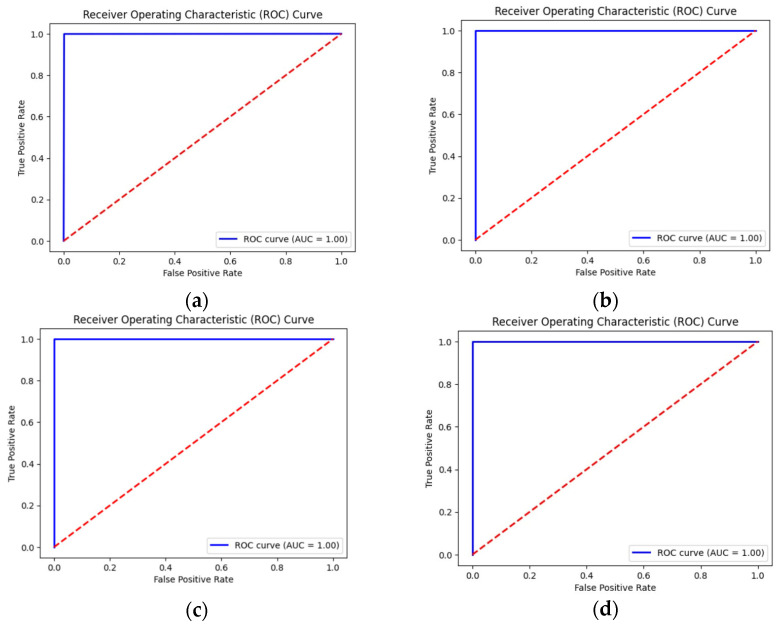
ROC curves for all data reduction for the downstream obtain from the Gaussian pretext model. (**a**) Using all the dataset; (**b**) 20 percent reduction; (**c**) 50 percent reduction; and (**d**) representing 70 percent reduction.

**Figure 14:**
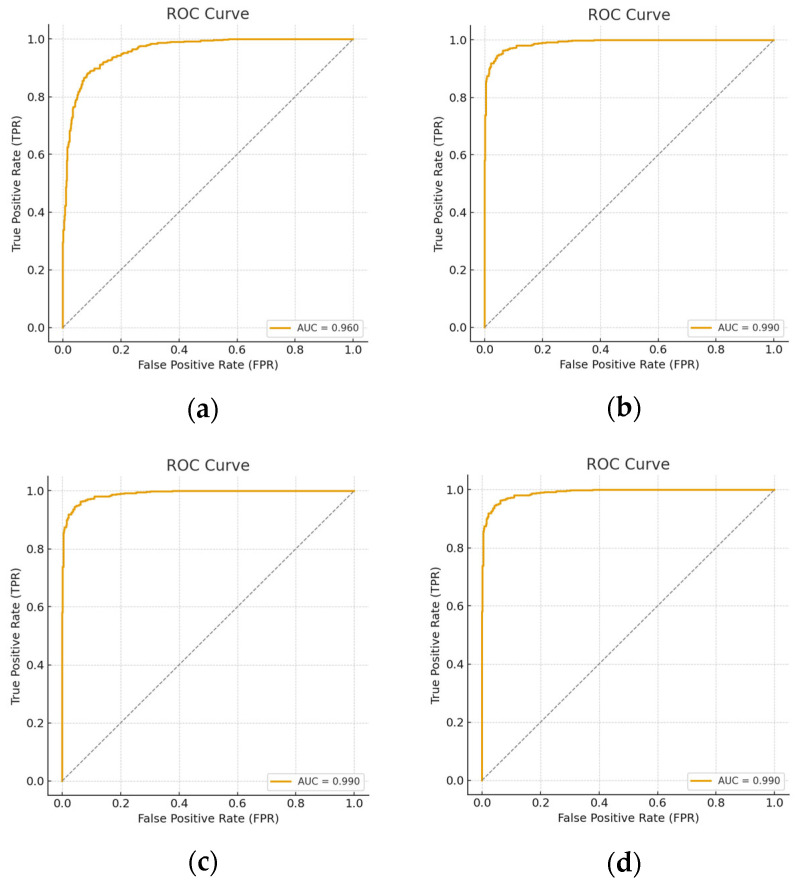
ROC curves for all data reduction for the downstream obtain from generative pretext model. (**a**) using 70% the dataset, (**b**) full dataset (**c**) 50% reduction and (**d**) 70% reduction.

In this section of the paper, AUC, ROC, precision, sensitivity, specificity, and F1-score were further evaluated on the three models across all data scenarios. Table 7 displays all the mentioned metrics. Figure 12, Figure 13 and Figure 14 represents the Receiver Operating Characteristic (ROC) curve obtained from the downstream task of the rotation model, Gaussian model and generative model under all reduction scenarios, respectively. The performance of the downstream tasks is more influenced by their respective pretext models, indicating that well-learned features were extracted from the unannotated data.

Table 7 demonstrates that the Gaussian noise pretext model achieved nearly perfect performance across all metrics, with AUC consistently equal to 1, even under data reduction. The rotation model showed strong initial accuracy but degraded sharply at 50% and 70% reductions, reflecting weak robustness. The generative model maintained high accuracy and balanced metrics but revealed more sensitivity to data scarcity and perturbations than Gaussian noise.

To fully evaluate data reduction in self-supervised learning and determine whether overfitting or underfitting is present, the proposed method was tested using a second dataset called Ohio State Cardiac MRI Raw Data (OCMR), which is a relatively more challenging dataset. According to this experience, Table 8 shows more moderate results than Table 7. Gaussian noise still performed best, with stable sensitivity, specificity, and AUC (~0.94–0.95) across reductions. Rotation was weaker, especially at 50% reduction, where precision and AUC dropped. The generative model remained competitive, maintaining good balance between sensitivity and precision, though with lower robustness at higher reductions. Overall, all models performed worse than in Table 7, reflecting dataset variability.

While Table 7 shows near-perfect performance on the CAD dataset, Table 8 reveals performance drops on OCMR, highlighting generalization challenges. Gaussian noise consistently outperformed others in both datasets, demonstrating superior robustness and stability. Rotation was the least reliable across both datasets, while the generative model showed moderate but consistent performance. This comparison confirms Gaussian noise as the most effective pretext task for cross-dataset CAD MRI classification.

### 4.2. Statistical Analysis of the Results

In our study, we investigated the statistical significance of the results using one-way ANOVA with Tukey’s HSD, employing various percentages of the dataset as independent variables. The ANOVA test is a parametric statistical significance test employed to ascertain the impact of an independent variable on a dependent variable and to assess differences by comparing the variance of groups with the variance within groups [32]. Upon detecting a statistically significant difference with the ANOVA test, Tukey’s HSD is employed to ascertain the specific groups between which this difference exists. Tukey’s HSD evaluates pairwise differences and determines the presence of statistically significant differences between two distinct groups, utilizing confidence intervals and *p*-values. The ANOVA test outcomes for the evaluation measures derived from our investigation are displayed in Table 9. Table 10 presents the statistical pairwise comparison analysis for the evaluation metrics.

The ANOVA test yielded a F value of 32.1776 and a *p* value of less than 0.00001, indicating statistical significance at the *p* < 0.05 level. The ANOVA test performed in the study indicates significant differences among the percentages of the dataset regarding several evaluation parameters (SD = 0.0176, df = 3, MS = 0.0069, *p* < 0.05). Subsequently, the Tukey HSD test evaluated the percentage pairs exhibiting significant differences. Notable disparities were observed in the B_1_:B_2_, B_2_:B_3_, and B_2_:B_4_ comparisons (*p* < 0.00000), establishing that the B_3_ percentage was statistically superior to the others. No significant differences were noted in the comparisons B_1_:B_3_ (*p* = 0.7415), B_1_:B_4_ (*p* = 0.0760), and B_3_:B_4_ (*p* = 0.3108). These findings validate that certain percentages exceed others on specific evaluation measures, and these disparities are statistically significant. The statistical methods employed in our work offer a robust foundation for percentage comparisons, rather than depending exclusively on raw accuracy metrics. Statistical significance tests prevent misinterpretations in percentage selection and tell researchers about the randomness of performance outcomes.

### 4.3. Limitations of the Study and Future Work

In this study, several limitations should be noted. First, CAD was treated as a binary classification problem, while clinical diagnosis typically involves multi-class grading of stenosis severity. Second, cardiac MRI is not the clinical gold standard for CAD diagnosis; coronary angiography or CT angiography are more commonly used. Third, the study was based on a single dataset, and external validation across multiple centers, scanners, and populations was not conducted. Fourth, only three SSL pretext approaches were evaluated, which limits the generalizability of the findings. Finally, multimodal data such as ECG, EEG, or hemodynamic signals were not incorporated, which could enhance the model’s real-world applicability. Only FGSM and PGD adversarial attacks were assessed, leaving uncertainty regarding model performance under other perturbations.

Future research should expand the set of SSL approaches to include recent methods such as BYOL, SwAV, MAE, contrastive predictive coding, and hybrid architectures (e.g., LSTM/U-Net) for a more comprehensive comparison. External validation on diverse patient cohorts and imaging centers will be essential for ensuring generalizability. Incorporating multimodal signals and temporal data could further improve model performance and support CAD assessment beyond imaging alone. Evaluating additional adversarial perturbations and exploring patient-specific optimization strategies [33] could enhance reliability and adaptability. Future work will also address a broader scope under adversarial attacks. While this study evaluates robustness using FGSM and PGD adversarial attacks in biomedical imaging and does not directly explore electronic circuit design, the efficient and robust feature representations obtained through SSL could inform future hardware implementations for low-power and real-time medical imaging systems. Leveraging SSL-based feature extraction to guide embedded medical device design under low-power and real-time constraints, as well as extending SSL methods to multimodal bio signals such as ECG and EEG to complement imaging-based CAD assessment, represents promising directions. Cross-domain comparisons with mechanical stress contexts were not conducted and represent an interesting avenue for future research to further explore model generalizability and although our study uses self-supervised learning to handle limited data, future work could explore optimized numerical methods to further improve performance under extreme data scarcity.

## 5. Conclusions

The study presents supervised and self-supervised pretext models for coronary artery disease (CAD) classification using cardiac MRI. The results show that well-designed pretext models can achieve high accuracy, even with limited training data. Among the models, those trained with Gaussian noise achieved the best performance in out-of-distribution (OOD) detection, showed notable resilience to adversarial perturbations under the used evaluation metrics. The generative pretext model maintained strong performance under similar conditions, whereas the rotation-based model performed relatively weaker. These findings highlight the critical role of pretext task selection in self-supervised learning (SSL) frameworks.

The choice of Gaussian noise, image rotation, and generative reconstruction as pretext tasks was guided by recent research trends and selected as representative methods for a focused comparison. However, a broader evaluation that includes more recent SSL techniques—such as BYOL, SwAV, MAE, contrastive predictive coding, and hybrid architectures (e.g., LSTM/U-Net)—would be necessary to draw more general conclusions about supervised versus unsupervised pretext approaches [34]. FEM–AI integration has also been suggested as a promising strategy for device-assisted monitoring and rehabilitation, complementing imaging-based SSL approaches [35].

Overall, this study demonstrates that careful selection of pretext tasks can produce high-performing models even in data-limited scenarios. While exploratory in nature, the findings provide insights into effective SSL design for medical imaging and outline key directions for future research to improve clinical applicability and generalizability.

## Figures and Tables

**Table 1:** Pseudo-labels used.

Literature	Description	Generation of Pseudo-Label
[16]	Arrangement of image segments and tasking the pretext model to predict the correct order	Pseudo-labels for the correct puzzles
[17]	Rotates the images 90°, 180°, etc. The model is trained to predict the rotation.	Labels are from the rotated angles
[14]	Uses image augmentation. The pretext is to differentiate between the original image and the augmented images	Label from the original images and that of the augmented images
[18]	This approach uses clustering, and the pretext model is trained with a classification-based objective.	Pseudo-labels are obtained from the clustering

**Table 2:** Without the use of pseudo-labels.

Literature	Description	Generation of Label or Not
[19]	Reconstruction of images	Direct from the original images no label needed
[20,21]	Similarities between images with Moco having a memory bank for negative samples	Based on contrastive learning and contrastive loss, respectively
[22]	Predicting missing part	Reconstruction of missing pixels
[23]	Learns features without negative sample only based on self-distillation	No pseudo-labels needed

**Table 3:** Downstream from Gaussian noise pretext.

% Reduction	Training Accuracy	Val Accuracy	Training Loss	Val Loss	PGD AND FGSM Attack	OOD
None	0.989	0.988	0.0019	0.0014	No effect	0.001
20	0.990	0.998	0,07	0.04	No effect	0.005
50	0.999	0.999	0.08	0.014	No effect	0.04
70	0.999	0.999	0.05	0.47	Less effect	0.4

**Table 4:** Downstream from rotation pretext.

Percentage	Training Accuracy	Val Accuracy	Training Loss	Val Loss	PGD Attack and FGSM Attack	OOD
None	0.99	0.99	0.02	0.015	No effect	0.01
20	0.97	0.98	0.04	0.098	No effect	0.1
50	0.84	0.80	0.5	0.6	There is an effect	0.34
70	0.70	0.72	0.6	0.7	There is an effect	0.49

**Table 5:** Downstream from generative pretext.

Percentage	Training Accuracy	Val Accuracy	Training Loss	Val Loss	PGD Attack and FGSM Attack	OOD
None	0.96	0.95	0.017	0.001	No effect	0.0015
20	0.95	0.96	0.12	0.025	No effect	0.05
50	0.82	0.84	0.25	0.21	Less effect	0.3
70	0.77	0.715	0.5	0.1	There is an effect	0.40

**Table 6:** Gaussian noise, rotation, generative vs. SimCLR comparative analysis.

Method	Pretext	Best Val Ac (50%)	PGD Attack and FGSM Attack	OOD
Gaussian Noise	supervised	99.9	Highly robust	0.04
Rotation	Supervised	80	Poor robustness beyond 20% data reduction	0.34
Generative	Unsupervised	84	Moderate robustness	0.30
SimCLR	Unsupervised	97.7	Moderate robustness	0.29

**Table 7:** Evaluation metrics for all three downstream tasks on the CAD Cardiac MRI dataset.

Model	Reduction	Sensitivity	Specificity	Precision	F-Score	AUC
GAUSE	Full	0.98	0.99	0.99	0.99	1
	50	0.98	0.98	0.99	0.98	1
	20	0.99	0.98	0.98	0.98	1
	70	0.97	0.96	0.97	0.98	1
ROTATION	Full	0.98	0.96	0.95	0.97	0.99
	50	0.97	0.75	0.79	0.81	0.87
	20	0.97	0.96	0.95	0.97	0.98
	70	0.95	0.94	0.96	0.90	0.85
GEN-MODEL	Full	0.97	0.97	0.96	0.96	0.99
	50	0.95	0.95	0.97	0.96	0.99
	20	0.96	0.94	0.93	0.95	0.99
	70	0.99	0.90	0.97	0.90	0.96

**Table 8:** Evaluation metrics for all three downstream tasks on the Ohio State Cardiac MRI Raw Data (OCMR).

Model	Reduction	Sensitivity	Specificity	Precision	F-Score	AUC
GAUSE	Full	0.94	0.94	0.93	0.94	0.95
	50	0.95	0.93	0.93	0.94	0.94
	20	0.94	0.94	0.93	0.93	0.95
	70	0.93	0.92	0.91	0.94	0.94
ROTATION	Full	0.93	0.93	0.90	0.93	0.93
	50	0.92	0.86	0.85	0.88	0.90
	20	0.94	0.91	0.92	0.91	0.93
	70	0.93	0.94	0.90	0.92	0.88
GEN-MODEL	Full	0.94	0.90	0.91	0.92	0.95
	50	0.93	0.92	0.91	0.93	0.94
	20	0.94	0.91	0.89	0.90	0.94
	70	0.95	0.88	0.92	0.88	0.92

**Table 9:** ANOVA test results for evaluation metrics obtained from test results for various percentages of the dataset used in experiments.

Source	SS	df	MS
Between-Evaluation Metric	0.0108	2	0.058
Within-Evaluation Metric	0.0025	18	0.0002
Total	0.0133	20	

**Table 10:** Statistical pairwise comparison analysis of different evaluation metrics.

		HSD_0.05_ = 0.0208 HSD_0.01_ = 0.0273	Q_0.05_ = 3.8576 Q_0.01_ = 4.7895
B_1_:B_2_	M_1_ = 0.98 M_2_ = 0.97	0.05	Q = 9.07 (*p* = 0.0000)
B_1_:B_3_	M_1_ = 0.98 M_3_ = 0.99	0.01	Q = 0.87 (*p* = 0.7415)
B_1_:B_4_	M_1_ = 0.98 M_4_ = 0.95	0.01	Q = 2.96 (*p* = 0.0760)
B_2_:B_3_	M_2_ = 0.97 M_3_ = 0.99	0.05	Q = 10.63 (*p* = 0.0000)
B_2_:B_4_	M_2_ = 0.97 M_4_ = 0.95	0.07	Q = 15.18 (*p* = 0.0000)
B_3_:B_4_	M_3_ = 0.99 M_4_ = 0.95	0.01	Q = 2.01 (*p* = 0.3108)

## Data Availability

The original data are included in the article. Further inquiries can be directed to the corresponding author.

## Abbreviations

The following abbreviations are used in this manuscript:

SSL	Self-Supervised Learning
OOD	Out-of-Distribution
PDG	Projected Gradient Descent
FGSM	Fast Gradient Sign Method
CAD	Coronary Artery Disease
